# Exposure to air pollution and non-neoplastic digestive system diseases: findings from the China health and retirement longitudinal study

**DOI:** 10.3389/fpubh.2024.1372156

**Published:** 2024-11-15

**Authors:** Yanqi Kou, Shenshen Du, Weiwei Du, Weixiang Ye, Yuping Yang, Ling Qin

**Affiliations:** ^1^The First Affiliated Hospital, College of Clinical Medicine of Henan University of Science and Technology, Luoyang, Henan, China; ^2^Department of Gastroenterology, Affiliated Hospital of Guangdong Medical University, Zhanjiang, China; ^3^Department of Gastroenterology, Huanghe Sanmenxia Hospital, Sanmenxia, Henan, China; ^4^Department of Cardiovascular Medicine, Huanghe Sanmenxia Hospital, Sanmenxia, Henan, China; ^5^Department of General Medicine, Dongpu Branch of The First Affiliated Hospital, Jinan University, Guangzhou, China

**Keywords:** digestive disease, air pollution, older adult, CHARLS, Chinese

## Abstract

**Objectives:**

Increasing concern about air pollution’s impact on public health underscores the need to understand its effects on non-neoplastic digestive system diseases (NNDSD). This study explores the link between air pollution and NNDSD in China.

**Methods:**

We conducted a national cross-sectional study using 2015 data from the China Health and Retirement Longitudinal Study (CHARLS), involving 13,046 Chinese adults aged 45 and above from 28 provinces. Satellite-based spatiotemporal models estimated participants’ exposure to ambient particulate matter (3-year average). An analysis of logistic regression models was conducted to estimate the association between air pollutants [particulate matter with a diameter ≤ 2.5 μm (PM_2.5_) or ≤10 μm (PM_10_), sulfur dioxide (SO_2_), nitrogen dioxide (NO_2_), ozone (O_3_), and carbon monoxide (CO)] and NNDSD. Interaction analyses were conducted to examine potential modifiers of these associations.

**Results:**

The prevalence of NNDSD among participants was 26.29%. After adjusted for multivariate factors, we observed a 6% [odd ratio (OR) = 1.06, 95% confidence interval (CI): 0.94, 1.19], 23% (OR = 1.23, 95% CI: 1.09, 1.38), 26% (OR = 1.26, 95% CI: 1.12, 1.41), 30% (OR = 1.30, 95% CI: 1.16, 1.46), 13% (OR = 1.13, 95% CI: 1.01, 1.27) and 27% (OR = 1.27, 95% CI: 1.13, 1.43) increase in NNDSD risk with an interquartile range increase in PM_2.5_ (23.36 μg/m^3^), PM_10_ (50.33 μg/m^3^), SO_2_ (17.27 μg/m^3^), NO_2_ (14.75 μg/m^3^), O_3_ (10.80 μg/m^3^), and CO (0.42 mg/m^3^), respectively. Interaction analyses showed that PM_2.5_, SO_2_, and O_3_ had stronger effects on NNDSD risk among older adults, highly educated individuals, smokers, and married people, respectively.

**Conclusion:**

This study demonstrates that long-term exposure to PM_2.5_, PM_10_, SO_2_, NO_2_, O_3_, and CO is positively associated with NNDSD risk in Chinese adults aged 45 and above. Implementing intervention strategies to enhance air quality is essential for reducing the burden of NNDSD.

## 1 Background

Non-neoplastic digestive system diseases (NNDSD) are non-cancerous digestive disorders, encompassing conditions such as gastroenteritis, gastroesophageal reflux disease (GERD), inflammatory bowel disease (IBD), irritable bowel syndrome (IBS), and liver diseases, among others (1, 2). There were 100,317 deaths related to non-malignant gastrointestinal diseases in the United States in 2019 (3). The incidence of digestive disorders has been increasing in China and other countries with Western societies (4). Research is increasingly revealing a pathogenic continuum between certain NNDSD and the emergence of malignant tumors of the gastrointestinal tract (5, 6). Millions worldwide suffer from these diseases, which also impose significant economic burdens, including high medical costs and lost work hours (7). Consequently, NNDSD represents a significant aspect of public health, and identifying risk factors for NNDSD is crucial for preventing and controlling NNDSD.

Industrialization, urbanization, and urban population growth have made air pollution one of the most critical environmental factors (8). Exposure to chronic air pollution [particulate matter with a diameter ≤ 2.5 μm (PM_2.5_) or ≤10 μm (PM_10_), sulfur dioxide (SO_2_), nitrogen dioxide (NO_2_), ozone (O_3_), and carbon monoxide (CO)] has been associated with respiratory diseases, cardiometabolic diseases, and cancer (9–12). Air pollution has been linked to digestive disorders such as enteritis, appendicitis, IBD, liver disease, and peptic ulcers. An association has been established between air pollution exposure and a higher incidence of NNDSD in urban populations (13–15). Our methodology extends previous research by incorporating a broader spectrum of air pollutants and a larger sample size. Prior studies primarily focused on PM_2.5_ and its impacts; our study included comprehensive pollutants such as PM_10_, SO_2_, NO_2_, O_3_, and CO, allowing for a more detailed analysis of air quality impacts on NNDSD. The effects of air pollution on digestive diseases have also been studied from a mechanistic perspective. Environmental exposure directly impacts epithelial cells, increases intestinal permeability, induces oxidative stress, triggers systemic inflammation, and alters gut microbiota (16–18). Despite their prevalence, the link between environmental factors, especially air pollution, and NNDSD remains largely unexplored. Particularly evident is this gap in China and other developing countries, where air pollution levels are high and large-scale population-based studies are lacking.

We conducted a national cross-sectional study to investigate the relationship between six major air pollutants (PM_2.5_, PM_10_, SO_2_, NO_2_, O_3_, and CO) and NNDSD to develop feasible strategies for preventing and controlling the disease.

## 2 Methods

### 2.1 Study population

The China Health and Retirement Longitudinal Study (CHARLS) selected participants from a national cohort of residents aged 45 and older. To gather representative data for middle-aged and older Chinese people, the study used GIS software and multistage probability sampling across 28 provinces. In accordance with the Declaration of Helsinki, Peking University’s Ethics Review Board approved the CHARLS protocol (IRB00001052-11015). Informed consent was obtained from all participants.

CHARLS started in 2011 and has been conducted every 2–3 years since then. Evaluations of Chinese air pollution levels have predominantly been conducted since 2013. Based on the CHARLS dataset from 2015, we conducted a cross-sectional study of 21,095 middle-aged and older adult who underwent medical examinations. We excluded data with missing key information: age (*n* = 364), body mass index (*n* = 3,348), smoking status (*n* = 11), alcohol consumption (*n* = 9), health status (*n* = 307), marital status (*n* = 2), residence (*n* = 86), education level (*n* = 2,945), and participants under 45 years old (*n* = 977). These exclusions were necessary to maintain the integrity and the analytical robustness of our study findings. The study included 13,046 middle-aged and older adult individuals from 28 provinces in China (Supplementary Figure S1).

### 2.2 Definition of NNDSD

A physician-diagnosed NNDSD was identified as one of the outcomes of interest in the 2015 questionnaire. During the follow-up questionnaire, the self-report question (‘Have you been diagnosed by a physician with a gastric or other non-neoplastic disease of the digestive system?’) was used to determine if the patient had been diagnosed with NNDSD. A subject who answered ‘yes’ to this question was defined as having NNDSD.

### 2.3 Air pollution exposure assessment

Between 2013 and 2015, a geocoding system was employed to identify the residential addresses of study subjects. These geocoded addresses were then used by Artificial Intelligence (AI) to calculate ground-level concentrations of air pollutants such as PM_2.5_, PM_10_, SO_2_, NO_2_, O_3_ and CO for each individual. The data were derived from the ChinaHighAirPollutants (CHAP) dataset and were spatially resolved to 0.1° (approximately 10 km). To estimate ambient PM_2.5_, PM_10_, SO_2_, NO_2_, CO, and O_3_, surface measurements, remote sensing products, atmospheric reanalysis and model simulations were used. With surface measurements, a 10-fold cross-validation was performed to determine *R*^2^ and the root mean square error (RMSE) for all daily mean estimates of all air pollutants (Supplementary Table S1). Annual air pollution exposures were calculated based on each participant’s county-level home address. For the main effects analyses, we calculated each participant’s long-term air pollution exposure as the 3-year average of PM_2.5_, PM_10_, SO_2_, NO_2_, O_3_, and CO levels before the 2015 CHARLS survey. For sensitivity analyses, we used the two-year average air pollution concentrations.

### 2.4 Covariates

Covariate data from CHARLS 2015 were used to prepare this study. As a demographic covariate, age ranges (45–60 years, ≥60 years), gender (male, female), residence (rural or urban), educational level (primary school and below, secondary school or above), marital status (married/cohabiting, unmarried, separated/divorced/widowed). The variables of health behavior included Body Mass Index (BMI) (underweight, normal weight, overweight/obese), smoking status (no or yes), alcohol consumption status (no or yes), and health status (good/very good, fair, poor/very poor).

### 2.5 Statistical analyses

We used descriptive statistics to compare the basic characteristics of participants, presenting categorical variables as numbers (percentages). Differences between non-NNDSD and NNDSD participants were assessed using the Chi-squared test for independence. The strength and direction of the association between air pollutants were quantified using Pearson’s correlation coefficients, which measure linear correlations between variables (Supplementary Table S2).

Logistic regression was used to examine the impact of 3-year average air pollutant concentrations on NNDSD. We first developed a crude model without adjustments. The base model adjusted for five covariates (gender, age, residence, education level, marital status), and the main model was fully adjusted for additional covariates (smoking, drinking, BMI, health status). NNDSD was expressed as odds ratios (OR) for each increment of the interquartile range (IQR) in air pollutants with corresponding 95% confidence interval (CI). Subgroup analyses and interaction tests were conducted within the logistic regression framework to explore potential modifications of the air pollution effects on NNDSD by various factors, including sex, age, place of residence, education level, marital status, smoking, alcohol consumption, BMI, and health status. Interaction terms were added to the models to statistically test for these effects.

To verify the robustness of our results, we performed sensitivity analyses. First, we reran the analyses using the average air pollution concentrations for the 2 years before 2015. Second, we excluded participants who self-reported poor or very poor health. We also conducted propensity score matching (PSM) using a nearest neighbor ratio of 1:1 without replacement and a caliper width of 0.05. We conducted the analyses using SPSS Statistics 26, R software (version 4.2.2), and Python3.11. R was used for spatial data analysis, statistical modeling, and generating forest plots, with packages like ggplot2, plyr, maptools, forestplot, and matchit. Python was used for air pollution mapping, employing libraries such as geopandas, matplotlib.pyplot, osgeo, and gdal. Statistical significance was determined by a two-tailed *p-*value of less than 0.05.

## 3 Results

### 3.1 Characteristics of study participants

**Figure 1:**
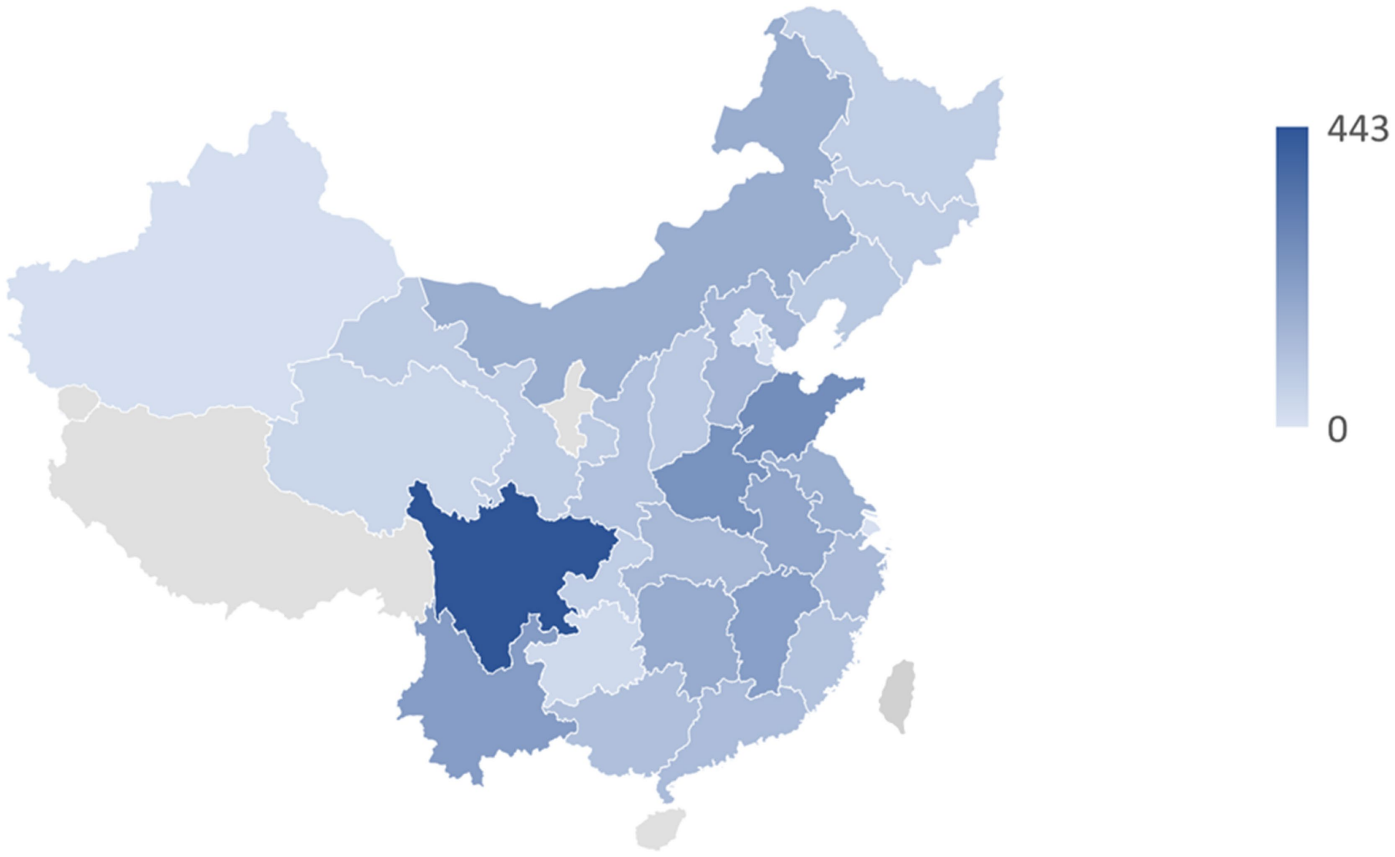
The geographical distribution of 13,046 middle-aged and older participants in 28 provinces of China.

In total, 13,046 participants were enrolled in this study, and the flowchart of participant enrollment can be seen in Supplementary Figure S1. Based on 13,046 participants from 28 provinces in China, Figure 1 illustrates their distribution. A summary of the basic characteristics of the study participants is provided in Table 1. The incidence rate of NNDSD was 26.29%. About 56.52% of the NNDSD cases were over 60 years old, and 53.18% of these were female. The majority of participants with NNDSD were married or cohabiting. Additionally, 26.81% of NNDSD cases were in poor health. There were 75.40% of participants who lived in rural areas and 68.70% who had only a primary school education or less. Aside from age and marital status, there was a significant difference between non-NNDSD and NNDSD patients with respect to sociodemographic and health behavioral characteristics.

**Table 1:** Basic characteristics of participants.

Characteristics	Total (*n* = 13,046)	NNDSD (*n* = 3,430)	Non-NNDSD (*n* = 9,616)	*P*
Age, *n* (%)				0.351
45–60	5,672 (43.48)	1,468 (42.80)	4,204 (43.72)	
≥60	7,374 (56.52)	1962 (57.20)	5,412 (56.28)	
Gender, *n* (%)				<0.001^***^
Males	6,108 (46.82)	1,450 (42.27)	4,658 (48.44)	
Females	6,938 (53.18)	1980 (57.73)	4,958 (51.56)	
Residence, *n* (%)				<0.001^***^
Rural	9,837 (75.40)	2,708 (78.95)	7,129 (74.14)	
Urban	3,209 (24.60)	722 (21.05)	2,487 (25.86)	
Marital status, *n* (%)				0.899
Married and living with a spouse	10,710 (82.09)	2,809 (81.90)	7,901 (82.17)	
Married but living without a spouse	599 (4.59)	162 (4.72)	437 (4.54)	
Single, divorced, and windowed	1737 (13.31)	459 (13.38)	1,278 (13.29)	
Education level, *n* (%)				<0.001^***^
Elementary school or below	8,962 (68.70)	2,550 (74.34)	6,412 (66.68)	
Middle school or above	4,084 (31.30)	880 (25.66)	3,204 (33.32)	
BMI, *n* (%)				<0.001^***^
<18.5	797 (6.11)	271 (7.90)	526 (5.47)	
18.5–23.9	6,289 (48.21)	1751 (51.05)	4,538 (47.19)	
≥24	5,960 (45.68)	1,408 (41.05)	4,552 (47.34)	
Smoking, *n* (%)				<0.001^***^
Yes	5,731 (43.93)	1,411 (41.14)	4,320 (44.93)	
No	7,315 (56.07)	2019 (58.86)	5,296 (55.07)	
Drinking, *n* (%)				<0.001^***^
Yes	4,479 (34.33)	1,060 (30.90)	3,419 (35.56)	
No	8,567 (65.67)	2,370 (69.10)	6,197 (64.44)	
Health status, *n* (%)				<0.001^***^
Good/very good	2,886 (22.12)	446 (13.00)	2,440 (25.37)	
Fair	6,663 (51.07)	1719 (50.12)	4,944 (51.41)	
Poor/very poor	3,497 (26.81)	1,265 (36.88)	2,232 (23.21)	

BMI, body mass index (calculated as weight in kilograms divided by height in meters squared); NNDSD, non-neoplastic digestive system diseases. *P < 0.05, **P < 0.01, ***P < 0.001.

**Figure 2:**
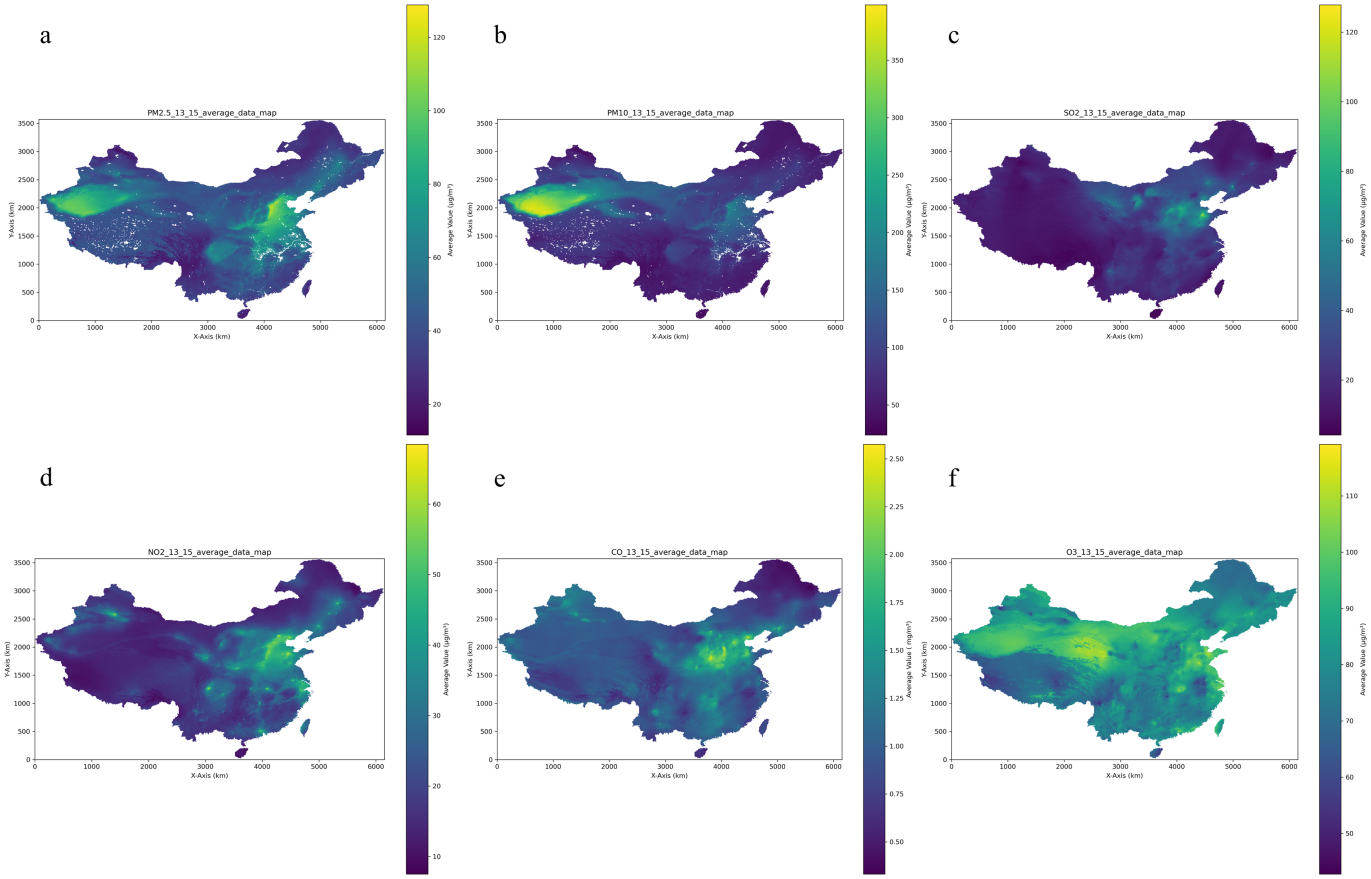
Air pollution distribution over 13–15 years of follow-up. **(a)** PM_2.5_: particle with aerodynamic diameter ≤ 2.5 μm; **(b)** PM_10_: particle with aerodynamic diameter ≤ 10 μm; **(c)** SO_2_: sulfur dioxide; **(d)** NO_2_: nitrogen dioxide; **(e)** CO: carbon monoxide; **(f)** O_3_: ozone.

A three-year average ambient concentration of PM_2.5_, PM_10_, SO_2_, NO_2_, O_3_, and CO for the six air pollutants is shown in Table 2 and Figure 2 at 49.54 ± 17.49 μg/m^3^ for PM_2.5_, 95.64 ± 33.71 μg/m^3^ for PM_10_, 31.13 ± 14.42 μg/m^3^ for SO_2_, 29.89 ± 9.51 μg/m^3^ for NO_2_, 83.04 ± 7.82 μg/m^3^ for O_3_ and 1.18 ± 0.33 mg/m^3^ for CO. Additionally, Pearson correlation analysis revealed strong correlations among several pollutants. Specifically, PM_2.5_ and PM_10_ were the most strongly correlated (*r* = 0.89), indicative of their common sources or similar atmospheric behaviors. Notably strong correlations were also observed between NO_2_ and PM_2.5_ (*r* = 0.86), as well as between CO and SO_2_ (*r* = 0.82). The range of correlation coefficients between the major pollutants (PM_2.5_, PM_10_, SO_2_, NO_2_, O_3_, and CO) varied from 0.70 to 0.89. O_3_ showed a distinct pattern with lower correlation coefficients compared to other pollutants, as shown in Supplementary Table S2.

**Table 2:** Descriptive statistics 3-years average levels of air pollution.

Variables	Mean	SD	P25	P50	P75	IQR
PM_2.5_ (μg/m^3^)	49.54	17.49	36.70	48.05	60.06	23.36
PM_10_ (μg/m^3^)	95.64	33.71	63.78	93.96	114.11	50.33
SO_2_ (μg/m^3^)	31.13	14.42	20.81	26.01	38.07	17.27
NO_2_ (μg/m^3^)	29.89	9.51	22.40	28.57	37.15	14.75
O_3_ (μg/m^3^)	83.04	7.82	77.00	81.11	87.81	10.80
CO (mg/m^3^)	1.18	0.33	0.94	1.08	1.36	0.42

PM_2.5_, particle with aerodynamic diameter ≤ 2.5 μm; PM_10_, particle with aerodynamic diameter ≤ 10 μm; SO_2_, sulfur dioxide; NO2, nitrogen dioxide; O_3_, ozone; CO, carbonic oxide; SD, standard deviation; P25, P50, P75, Lower, median and upper quartiles of variables; IQR, interquartile range.

### 3.2 Associations between air pollutants and NNDSD

**Figure 3:**
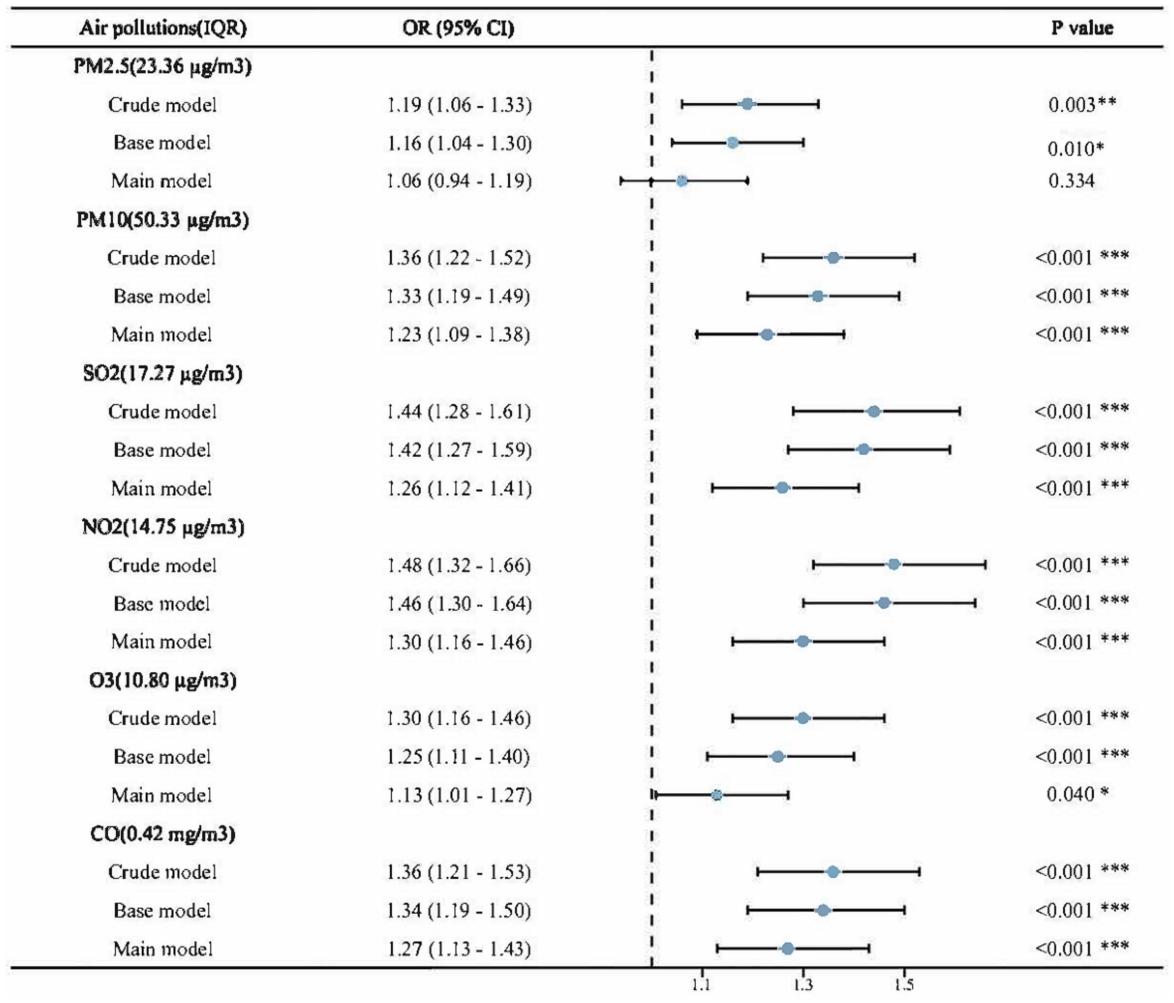
Associations between air pollution and NNDSD, per IQR increment in air pollutants. Crude model: no adjustment; Base model: Adjusted for age, gender, education level, marital status and residence; Main model: Base model + smoking, drinking, body mass index and health status. PM_2.5,_ particle with aerodynamic diameter ≤ 2.5 μm; PM_10_, particle with aerodynamic diameter ≤ 10 μm; SO_2_, sulfur dioxide; NO_2,_ nitrogen dioxide; CO, carbonic oxide; O_3_, ozone; NNDSD, non-neoplastic digestive system diseases; IQR, interquartile range; OR, odds ratios; CI, confidence interval. ^*^p < 0.05, ^**^p < 0.01, ^***^p < 0.001.

The association between each air pollutant and NNDSD is shown in Figure 3 and Supplementary Table S3. Higher levels of exposure to air pollutants (PM_2.5_, PM_10_, SO_2_, NO_2_, O_3_, and CO) are associated with an increased risk of NNDSD. According to the crude model, for each additional exposure IQR of PM_2.5_, PM_10_, SO_2_, NO_2_, O_3_ and CO, the OR for NNDSD was 1.19 (95%CI: 1.06, 1.33), 1.36 (95%CI:1.22, 1.52), 1.44 (95%CI: 1.28, 1.61), 1.48 (95%CI: 1.32, 1.66), 1.30 (95%CI: 1.16, 1.46), and 1.36 (95%CI: 1.21, 1.53). Significant associations in the base model persisted after adjusting for demographic covariates. Taking into account all covariates, including age, sex, place of residence, education, marital status, body mass index, smoking, drinking, and health status, we observed a 6% (fully adjusted OR = 1.06, 95%CI: 0.94, 1.19), 23% (fully adjusted OR = 1.23, 95%CI: 1.09, 1.38), 26% (fully adjusted OR = 1.26, 95%CI: 1.12, 1.41), 30% (fully adjusted OR = 1.30, 95%CI: 1.16, 1.46), 13% (fully adjusted OR = 1.13, 95%CI: 1.01, 1.27) and 27% (fully adjusted OR = 1.27, 95%CI: 1.13, 1.43) increase in NNDSD risk with a IQR increase in PM_2.5_, PM_10_, SO_2_, NO_2_, O_3_, and CO exposure in the main model, respectively. PM_2.5_’s *p*-value in the main model is 0.34(>0.05), and the rest <0.05.

### 3.3 Subgroup and interaction analyses of air pollutants and NNDSD

**Figure 4:**
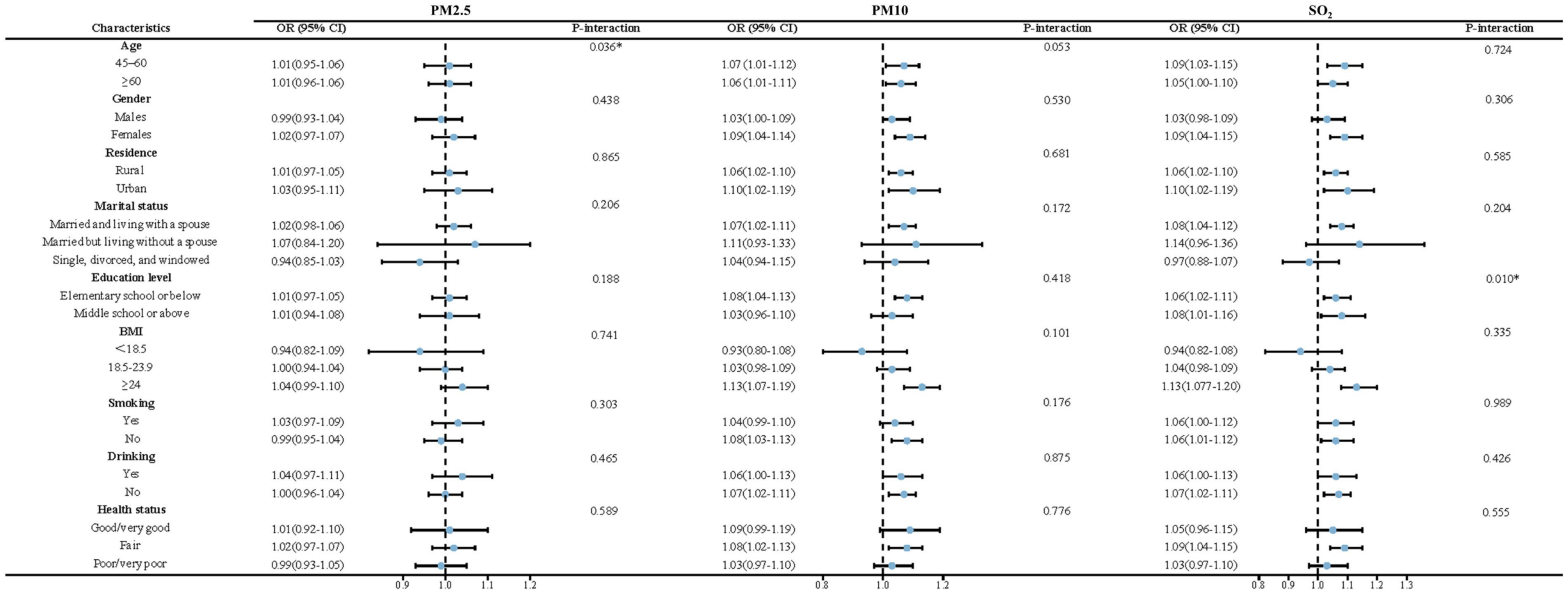
Subgroup and interaction analyses of air pollutants (PM_2.5_, PM_10_, and SO_2_) and NNDSD.

**Figure 5:**
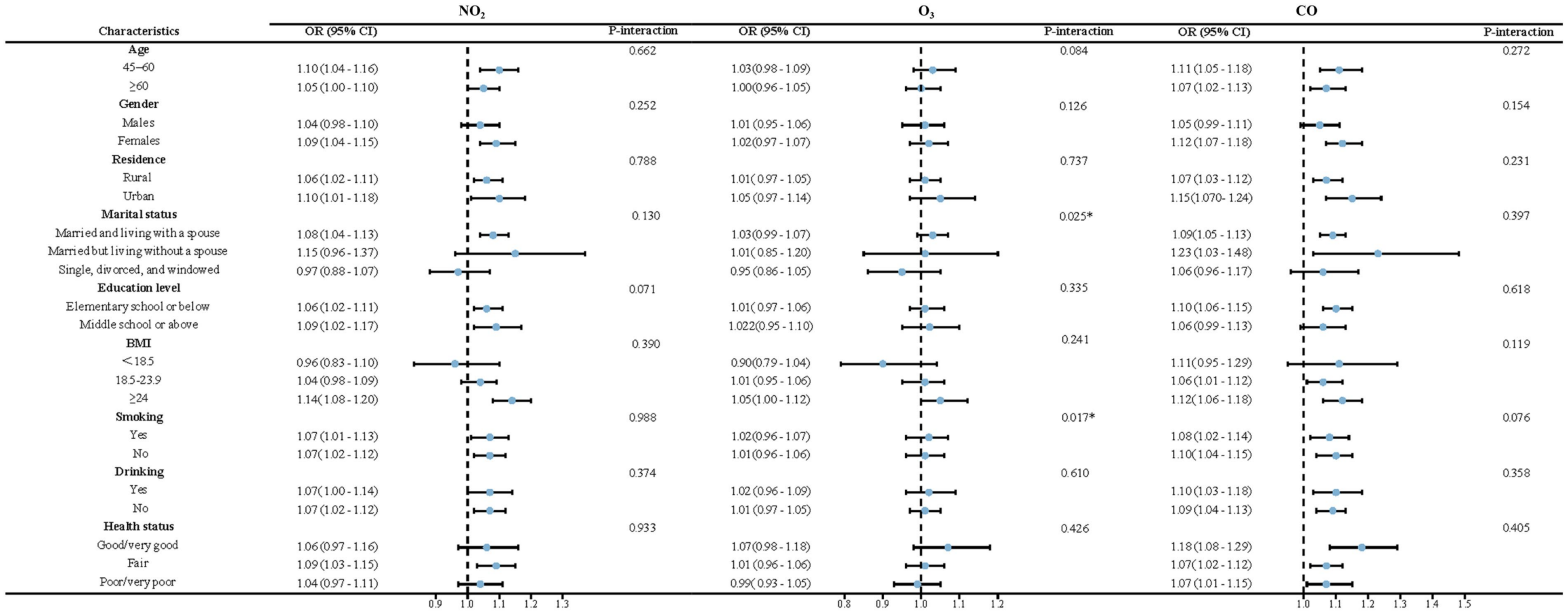
Subgroup and interaction analyses of air pollutants (NO_2_, O_3_, and CO) and NNDSD.

The results of the subgroup and interaction analyses are presented in Figures 4, 5. According to our findings, the following factors may modify the association between air pollution and NNDSD: PM_2.5_ significantly increased NNDSD risk in participants over 60 years of age (OR = 1.01, 95%CI: 0.96, 1.06), compared to participants aged 45–60 years (OR = 1.01, 95%CI: 0.95, 1.06) (*P* interaction = 0.04). In participants with higher education (OR = 1.08, 95%CI: 1.01, 1.16) as compared to those with lower education (OR = 1.06, 95%CI: 1.02, 1.11), SO_2_ had a greater effect on NNDSD in participants with higher education (*P* interaction = 0.01). In the group of smokers (OR = 1.02, 95%CI: 0.96, 1.07) (*P* interaction = 0.02) and of married or cohabiting couples (OR = 1.03, 95%CI: 0.99, 1.07) (*P* interaction = 0.03), ambient O_3_ had a greater effect on the rise in NNDSD.

### 3.4 Sensitivity analyses

The sensitivity analyses for the association between air pollution and NNDSD consistently demonstrated statistical significance in line with the main results. When the mean exposure concentration of air pollutants was reduced from 3 to 2 years, we observed that all the above significant associations remained strong (Supplementary Table S4). Additionally, we conducted sensitivity analyses that excluded poor/very poor health, which showed effect sizes and statistical significance similar to the main results, except for the association between exposure to O_3_ and NNDSD (Supplementary Table S5). In order to eliminate other potential confounders, sensitivity analyses were conducted when a 1:1 PSM was performed. Lastly, 3,430 participants with NNDSD were paired with the same number of participants non-NNDSD, and there were no significant differences in baseline characteristics between the two groups (Supplementary Table S7). Overall, these sensitivity analyses indicated that the main analysis results were robust (Supplementary Table S6).

## 4 Discussion

This study was one of the few national epidemiologic studies in China that investigated the relationship between continuous exposure to air pollutants and NNDSD. The purpose of this study was to investigate the effects of PM_2.5_, PM_10_, SO_2_, NO_2_, O_3_, and CO on NNDSD among middle-aged and older adult Chinese residents. Our findings revealed significant associations between several common air pollutants and the risk of NNDSD, with varying strengths across different pollutants. Nearly the entire global population is exposed to airborne pollution, recognized as a significant environmental health risk (19). In terms of policymaking aimed at protecting the public health from air pollution, our findings supported the hypothesis that air pollution exposure adversely affects NNDSD.

PM_2.5_ and PM_10_ are particulate air pollutants. Our study revealed that PM_2.5_ and PM_10_ demonstrate the strongest correlation in Pearson correlation analysis. Current research established a significant association between PM_10_ and NNDSD, persisting even after adjustment for confounding factors. Conversely, PM_2.5_ showed significant associations only in crude and base models, failing to maintain statistical significance in the main model. There appeared to be a difference in impact between air pollution particles of different sizes on digestive system health, with PM_10_ having a more pronounced effect in our study population than PM_2.5_.

This observation was corroborated by prior research. A comprehensive cross-sectional analysis involving 90,086 participants in southwest China demonstrated a positive association between prolonged exposure to environmental particulate matter and metabolically associated fatty liver disease. Additionally, animal studies have identified a steatohepatitis-like phenotype and liver fibrosis associated with such exposures, reinforcing the broader implications of particulate pollutants on liver health (20, 21). According to a cross-sectional study conducted in the United States, hospitalized patients with higher ambient PM_2.5_ exposure were more likely to develop non-alcoholic fatty liver disease (22). An extensive nationwide time-series study examined the adverse effects of short-term PM_2.5_ exposure on various digestive disorders, including intestinal infections, esophageal disease, gastritis, appendicitis, liver disease, gastrointestinal bleeding, and non-infectious gastroenteritis (23). On cooler days, there has been a significant increase in peptic ulcer hospitalizations in Taipei with rising levels of PM_10_ (24).

Research indicated that the impact of particulate air pollutants on the digestive system was primarily mediated through the ingestion of contaminants in food and water, as well as the direct effects of gaseous pollutants on the gastrointestinal tract during swallowing (25, 26). The deposition of air pollution particles in the lungs and the resultant epithelial cell responses, including the production of free radicals and inflammation, lead to the disruption of the alveolar barrier. These factors can subsequently enter the bloodstream, directly affecting intestinal epithelial cells and increasing intestinal permeability (27–30). Additionally, exposure to PM_2.5_ was associated with elevated levels of cluster of differentiation and systemic inflammation (31, 32). Animal studies demonstrate that PM_10_ exposure in mice leads to elevated pro-inflammatory cytokine levels in the small and large intestines, potentially stimulating alveolar macrophages to produce inflammatory factors present in the bloodstream during air pollution episodes (33, 34). Furthermore, studies have found that particulate air pollutants significantly altered the gut microbiota composition in both animals and humans, potentially increasing susceptibility to mucosal inflammation (17, 28, 30, 35).

We found a close association between SO_2_ levels and an increased risk of NNDSD. In subgroup and interaction analysis, we observed that the impact of environmental air pollutant SO_2_ on NNDSD was more pronounced among subjects with higher education. Residents near the Madin Dam in the State of Mexico showed a significant correlation between chronic exposure to SO₂ and liver function impairment. Specifically, individuals exposed to high levels of SO₂ exhibit elevated levels of lipid peroxidation products and oxidative stress markers in their blood, which are biomarkers indicating hepatocellular damage and dysfunction (36). In a study involving more than 2.7 million adults in Northwest China, each 10 μg/m^3^ increase in SO₂ concentration was associated with a 2.7-fold increase in the incidence risk of metabolically associated fatty liver disease (37). The increase in SO₂ levels have exacerbated inflammation and oxidative stress in the liver, both key drivers in the development of metabolically associated fatty liver disease. Research on pregnant women found that an increase in environmental SO₂ levels during the second trimester was associated with an increased risk of intrahepatic cholestasis of pregnancy (38). Long-term exposure to SO₂ may have increased pro-inflammatory cytokines such as IL-6 and IL-1β, and decreased hepatic bile transport proteins, leading to the accumulation of bile acids in the liver (39, 40).

A significant association between NO_2_ levels and NNDSD was observed. In our main model, an increase in NO_2_ exposure by one IQR was associated with a 30% increase in risk. An analysis of 8,566 older adult cases recorded between 2005 and 2010 by Tian et al. (41) found that a short-term increase in ambient NO_2_ levels may have increased the risk of peptic ulcer bleeding and related hospitalizations in Hong Kong’s older adult population. In a large-scale cohort study that investigated the associations between air pollutants and the risk of 12 gastrointestinal diseases, positive correlations were observed between NO_2_ and NOx with the risk of peptic ulcers and chronic gastritis (42). According to other studies, long-term exposures to NO_2_ were associated with ulcerative colitis but not Crohn’s disease (43). Research involving 329,048 adults in Taiwan and Hong Kong between 2001 and 2018 demonstrated that rising concentrations of NO₂ were linked to an increased risk of Non-Alcoholic Fatty Liver Disease (NAFLD) and its associated advanced fibrosis (44). Empirical evidence on the relationship between NO_2_ and digestive system diseases remained limited. As a component of traffic-related air pollution, NO_2_ was also found to be associated with elevated levels of cytokeratin-18, which may be linked to liver damage (45). NO_2_ swallowed through belching may trigger a nitrification reaction in the stomach, leading to redox interactions that disrupt the intestinal lining and promote gastrointestinal diseases (46).

Our study found that exposure to O_3_ is significantly associated with NNDSD, especially among smokers and those who are married or cohabiting. One research has shown that for every one standard deviation increase in O_3_ concentration, NAFLD decreases by 12%, and the incidence of advanced fibrosis decreases by 11%. This suggested that higher levels of O_3_ might have a protective effect against these conditions (44). Conversely, a study from Northwest China indicated that elevated O_3_ levels were linked to a slight increase in the risk of Metabolically Associated Fatty Liver Disease (37). Additionally, research from Korea reported a positive correlation between short-term exposure to O_3_ and elevated levels of gamma-glutamyl transferase (47). An analysis of the 7-day cumulative average of terrestrial O_3_ conducted by a Canadian researcher suggests that higher levels of environmental O_3_ exposure may increase the risk of perforated appendicitis (48). Kaplan’s findings indicated that higher 5-day average ozone levels increased appendicitis rates, especially in summer, with a greater impact on younger individuals than older adults. The specific mechanisms by which O_3_ influenced appendicitis incidence remained unclear, but inhaling or ingesting air pollutants likely triggered inflammatory responses in humans (49). Himuro (50) demonstrated that intrarectal administration of ozone gas causes transient epithelial cell damage, which is specifically caused by damage to DNA replication and cell cycle pathways. Exposure to O_3_ stimulated the production of tumor necrosis factors IL-6 and IL-8 and promotes systemic inflammation in humans, which may be a key factor in the pathogenesis of these diseases (51, 52).

In our study, exposure to CO was associated with an increased risk of NNDSD. There is limited literature on the impact of CO on digestive system disorders. Chinese researchers found that CO levels were significantly elevated on days of outpatient visits for enterocolitis (53). A study in Yichang, China, demonstrated that for every 1 mg/m^3^ increase in CO levels, there is a 19.04% (95% CI: 8.39, 29.68%) rise in daily outpatient visits for gastrointestinal diseases. From 2002 through 2007, Seo et al. (54) found that CO may increase the risk of GERD based on medical utilization data from the National Health Insurance Institute of Korea. Short-term exposure to CO air pollution was associated with decreased microvascular endothelial function (55). Microvascular dysfunction may have contributed to poor mucosal healing, refractory inflammatory ulcers, and intestinal injury in IBD patients, resulting in diminished vasodilatory capacity and inadequate tissue perfusion (56). CO’s high affinity for myoglobin may cause weakness in the lower esophageal sphincter, potentially leading to myasthenia gravis (57).

Our findings underscored the broader public health implications, suggesting that reducing air pollution levels could alleviate the burden of digestive system diseases among adults, particularly in areas with high pollution. Although many epidemiological studies link sustained air pollutant exposure to NNDSD risk, the underlying biological mechanisms remain unclear (58, 59). A biological and epidemiological study will be necessary to elucidate the mechanisms by which air pollution affects cognitive function in the future.

## 5 Limitations

There are several advantages to our study. Firstly, it is a nationwide study conducted in China, it offers a high level of generalizability. Secondly, this is the first study to investigate the relationship between air pollution exposure and NNDSD. Furthermore, we considered more air pollutants than previous studies, including the rarely considered NO_2_. However, we also have some limitations to our study. While we used satellite-based spatio-temporal modeling to estimate air pollution levels, exposure assessments were based on community locations, which may not fully represent individual exposures. Additionally, many individuals were excluded due to missing data on the variables, which may have led to a selection bias. Lastly, the findings may be affected by questionnaire and recall bias among older subjects. A lack of detailed types of NNDSD in this study also prevented a detailed study of life environmental factors on specific subtypes of NNDSD.

## 6 Conclusion

Our study suggests that chronic exposure to ambient air pollution may increase the prevalence of NNDSD among adults aged 45 and older in China. Particulates and gaseous pollutants like PM_2.5_, PM_10_, SO_2_, NO_2_, O_3_, and CO are significantly associated with NNDSD. This underscores the need for targeted air quality policies, which could reduce the NNDSD burden and prevent many cases.

## Data Availability

Publicly available datasets were analyzed in this study. This data can be found: https://charls.pku.edu.cn.
