# Supplementary material for: Exposure to air pollution and non-neoplastic digestive system diseases: findings from the China health and retirement longitudinal study
Source: Front Public Health. 2024 Nov 15;12:1372156. doi: 10.3389/fpubh.2024.1372156 (PMC11604608; doi:10.3389/fpubh.2024.1372156)
Supplement: Supplementary file 1 [file Data_Sheet_1.docx]

**Supplementary materials**

**
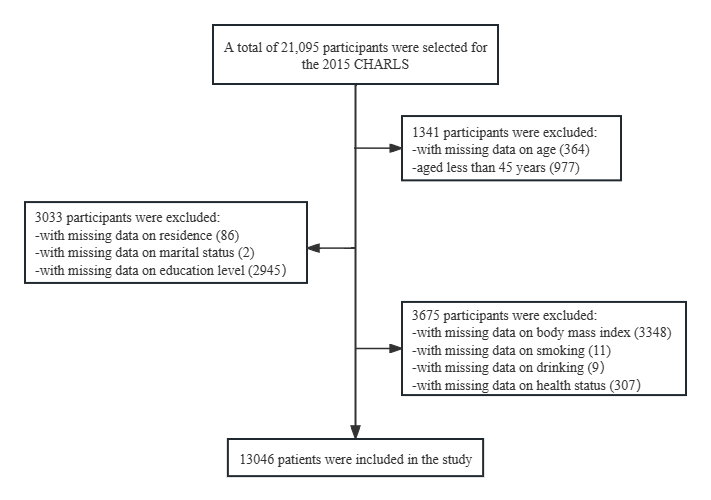
**

**Figure S1. The flowchart of participant selection.**

**Table S1. Air pollution estimation R2 and Root-Mean-Square Error based on 10-fold cross-validation**

| **Air pollutions** | PM_2.5_ | PM_10_ | SO_2_ | NO_2_ | O_3_ | CO |
| --- | --- | --- | --- | --- | --- | --- |
| R^2^ | 0.92 | 0.90 | 0.84 | 0.84 | 0.87 | 0.80 |
| Root-Mean-Square Error | 10.76 μg/m^3^ | 21.12 μg/m^3^ | 10.07 μg/m^3^ | 7.99 μg/m^3^ | 17.10 μg/m^3^ | 0.29 mg/m^3^ |

Abbreviations: PM_2.5,_ particle with aerodynamic diameter ≤2.5 μm; PM_10_, particle with aerodynamic diameter ≤10 μm; SO_2_, sulfur dioxide; NO_2,_ nitrogen dioxide; CO, carbonic oxide; O_3_, ozone.

| **Table S2. Person correlation coefficients of three-year average levels of air pollution** | | | | | | |
| --- | --- | --- | --- | --- | --- | --- |
| **Air pollutions** | PM_2.5_ | PM_10_ | SO_2_ | NO_2_ | O_3_ | CO |
| PM_2.5_ | 1 |  |  |  |  |  |
| PM_10_ | 0.891 | 1 |  |  |  |  |
| SO_2_ | 0.750 | 0.809 | 1 |  |  |  |
| NO_2_ | 0.858 | 0.809 | 0.797 | 1 |  |  |
| O_3_ | 0.451 | 0.489 | 0.468 | 0.575 | 1 |  |
| CO | 0.701 | 0.724 | 0.816 | 0.709 | 0.221 | 1 |

Abbreviations: PM_2.5,_ particle with aerodynamic diameter ≤2.5 μm; PM_10_, particle with aerodynamic diameter ≤10 μm; SO_2_, sulfur dioxide; NO_2,_ nitrogen dioxide; CO, carbonic oxide; O_3_, ozone;

**Table S3. Associations between air pollutants and prevalence of** **NNDSD**

|  | **Pollutants** | | **Crude model**  **OR and 95%CI** | ***P*** |  | **Base model**  **OR and 95%CI** | ***P*** |  | **Main model**  **OR and 95%CI** | ***P*** |
| --- | --- | --- | --- | --- | --- | --- | --- | --- | --- | --- |
|  | PM_2.5_ | 1.19 (1.06 - 1.33) | | 0.003 ^**^ |  | 1.16 (1.04 - 1.30) | 0.010 ^*^ |  | 1.06 (0.94 - 1.19) | 0.334 |
|  | PM_10_ | 1.36 (1.22 - 1.52) | | <0.001 ^***^ |  | 1.33 (1.19 - 1.49) | <0.001 ^***^ |  | 1.23 (1.09 - 1.38) | <0.001 ^***^ |
|  | SO_2_ | 1.44 (1.28 - 1.61) | | <0.001 ^***^ |  | 1.42 (1.27 - 1.59) | <0.001 ^***^ |  | 1.26 (1.12 - 1.41) | <0.001 ^***^ |
|  | NO_2_ | 1.48 (1.32 - 1.66) | | <0.001 ^***^ |  | 1.46 (1.30 - 1.64) | <0.001 ^***^ |  | 1.30 (1.16 - 1.46) | <0.001 ^***^ |
|  | O_3_ | 1.30 (1.16 - 1.46) | | <0.001 ^***^ |  | 1.25 (1.11 - 1.40) | <0.001 ^***^ |  | 1.13 (1.01 - 1.27) | 0.040 ^*^ |
|  | CO | 1.36 (1.21 - 1.53) | | <0.001 ^***^ |  | 1.34 (1.19 - 1.50) | <0.001 ^***^ |  | 1.27 (1.13 - 1.43) | <0.001 ^***^ |

Note: Abbreviations: PM_2.5,_ particle with aerodynamic diameter ≤2.5 μm; PM_10_, particle with aerodynamic diameter ≤10 μm; SO_2_, sulfur dioxide; NO_2,_ nitrogen dioxide; CO, carbonic oxide; O_3_, ozone; NNDSD, non-neoplastic digestive system diseases; OR, odds ratios; CI, confidence interval. ^*^ *P*<0.05; ^**^ *P*<0.01; ^***^*P*<0.001

Results were presented as OR and 95%CI of NNDSD per interquartile range increase in air pollution (PM_2.5_, 23.36 μg/m^3^; PM_10_, 50.33 μg/m^3^; SO_2_, 17.27 μg/m^3^; NO_2_, 14.75 μg/m^3^; O_3_, 10.80 μg/m^3^; CO, 0.42 mg/m^3^)

Crude model: no adjustment.

Base model: Adjusted for age, gender, education level, marital status and residence.

Main model: Base model + smoking, drinking, body mass index and health status

| Air pollutants |  | 2-year average | |  |  | 3-year average | |
| --- | --- | --- | --- | --- | --- | --- | --- |
|  |  | OR and 95%CI | *P* |  |  | OR and 95%CI | *P* |
| PM_2.5_ |  | 1.08 (0.96 - 1.21) | 0.178 |  |  | 1.06 (0.94 - 1.19) | 0.334 |
| PM_10_ |  | 1.22 (1.09 - 1.38) | <0.001 ^***^ | |  | 1.23 (1.09 - 1.38) | <0.001 ^***^ |
| SO_2_ |  | 1.25 (1.12 - 1.41) | <0.001 ^***^ |  |  | 1.26 (1.12 - 1.41) | <0.001 ^***^ |
| NO_2_ |  | 1.23 (1.10 - 1.38) | <0.001 ^***^ | |  | 1.30 (1.16 - 1.46) | 0.040 ^*^ |
| O_3_ |  | 1.13 (1.01 - 1.27) | 0.046 ^*^ | |  | 1.13 (1.01 - 1.27) | <0.001 ^***^ |
| CO |  | 1.27 (1.12 - 1.43) | <0.001 ^***^ | |  | 1.27 (1.13 - 1.43) | <0.001 ^***^ |

**Table S4. Sensitivity analysis of the association between air pollution and NNDSD using two-year average concentrations of air pollutants**

Abbreviations: PM_2.5,_ particle with aerodynamic diameter ≤2.5 μm; PM_10_, particle with aerodynamic diameter ≤10 μm; SO_2_, sulfur dioxide; NO_2,_ nitrogen dioxide; CO, carbonic oxide; O_3_, ozone; OR, odds ratios; CI, confidence interval.

^*^ *P*<0.05; ^**^ *P*<0.01; ^***^*P*<0.001.

**Table S5. Sensitivity analysis by excluding participants who in poor/very poor health in the association between air pollution and NNDS**

| Air pollutants | Participants who in poor/very poor health were excluded | |  | Participants who in poor/very poor health were included | |
| --- | --- | --- | --- | --- | --- |
|  | OR and 95%CI | *P* |  | OR and 95%CI | *P* |
| PM_2.5_ | 1.08 (0.94 - 1.25) | 0.284 |  | 1.06 (0.94 - 1.19) | 0.334 |
| PM_10_ | 1.29 (1.12 - 1.48) | <0.001 ^***^ | | 1.23 (1.09 - 1.38) | <0.001 ^***^ |
| SO_2_ | 1.31 (1.14 - 1.51) | <0.001 ^***^ |  | 1.26 (1.12 - 1.41) | <0.001 ^***^ |
| NO_2_ | 1.34 (1.16 - 1.55) | <0.001 ^***^ | | 1.30 (1.16 - 1.46) | 0.040 ^*^ |
| O_3_ | 1.14 (0.99 - 1.32) | 0.071 | | 1.13 (1.01 - 1.27) | <0.001 ^***^ |
| CO | 1.29 (1.12 - 1.49) | <0.001 ^***^ | | 1.27 (1.13 - 1.43) | <0.001 ^***^ |

Abbreviations: PM_2.5,_ particle with aerodynamic diameter ≤2.5 μm; PM_10_, particle with aerodynamic diameter ≤10 μm; SO_2_, sulfur dioxide; NO_2,_ nitrogen dioxide; CO, carbonic oxide; O_3_, ozone; OR, odds ratios; CI, confidence interval.

^*^ *P*<0.05; ^**^ *P*<0.01; ^***^*P*<0.001.

**Table S6. Sensitivity analysis of using** **propensity score matching in the association between air pollution and NNDSD**

| Air pollutants | post-propensity score matching | |  | pre-propensity score matching | |
| --- | --- | --- | --- | --- | --- |
|  | OR and 95%CI | *P* |  | OR and 95%CI | *P* |
| PM_2.5_ | 1.12 (0.98 - 1.28) | 0.105 |  | 1.06 (0.94 - 1.19) | 0.334 |
| PM_10_ | 1.28 (1.12 - 1.47) | <0.001 ^***^ | | 1.23 (1.09 - 1.38) | <0.001 ^***^ |
| SO_2_ | 1.36 (1.18 - 1.56) | <0.001 ^***^ |  | 1.26 (1.12 - 1.41) | <0.001 ^***^ |
| NO_2_ | 1.39 (1.21 - 1.60) | <0.001 ^***^ | | 1.30 (1.16 - 1.46) | 0.040 ^*^ |
| O_3_ | 1.19 (1.04 - 1.37) | 0.012^*^ | | 1.13 (1.01 - 1.27) | <0.001 ^***^ |
| CO | 1.38 (1.20 - 1.58) | <0.001 ^***^ | | 1.27 (1.13 - 1.43) | <0.001 ^***^ |

Abbreviations: PM_2.5,_ particle with aerodynamic diameter ≤2.5 μm; PM_10_, particle with aerodynamic diameter ≤10 μm; SO_2_, sulfur dioxide; NO_2,_ nitrogen dioxide; CO, carbonic oxide; O_3_, ozone; OR, odds ratios; CI, confidence interval.

^*^ *P*<0.05; ^**^ *P*<0.01; ^***^*P*<0.001.

**Table S7. Characteristics of study patients before and after propensity score matching (PSM)**

| Variable | Before PSM | | | | | | After PSM | | |
| --- | --- | --- | --- | --- | --- | --- | --- | --- | --- |
|  | NNDSD  (n = 3430) | Non- NNDSD  (n = 9616) | *P* | SMD |  | NNDSD  (n = 3424) | Non- NNDSD  (n = 3424) | *P* | SMD |
| **Gender, n (%)** |  |  | **<.001** |  |  |  |  | 0.642 |  |
| Females | 1980 (57.73) | 4958 (51.56) |  | -0.123 |  | 1974 (57.65) | 1993 (58.21) |  | 0.011 |
| Males | 1450 (42.27) | 4658 (48.44) |  | 0.123 |  | 1450 (42.35) | 1431 (41.79) |  | -0.011 |
| **Age, n (%)** |  |  | 0.351 |  |  |  |  | 0.714 |  |
| 45–60 | 1468 (42.80) | 4204 (43.72) |  | 0.019 |  | 1462 (42.70) | 1477 (43.14) |  | 0.009 |
| ≥60 | 1962 (57.20) | 5412 (56.28) |  | -0.019 |  | 1962 (57.30) | 1947 (56.86) |  | -0.009 |
| **Residence, n (%)** |  |  | **<.001** |  |  |  |  | 0.462 |  |
| Rural | 2708 (78.95) | 7129 (74.14) |  | -0.110 |  | 2703 (78.94) | 2678 (78.21) |  | -0.018 |
| Urban | 722 (21.05) | 2487 (25.86) |  | 0.110 |  | 721 (21.06) | 746 (21.79) |  | 0.018 |
| **Marital status, n (%)** |  |  | 0.899 |  |  |  |  | 0.982 |  |
| Married and living with a spouse | 2809 (81.90) | 7901 (82.17) |  | 0.007 |  | 2805 (81.92) | 2811 (82.10) |  | 0.005 |
| Married but living without a spouse | 162 (4.72) | 437 (4.54) |  | -0.009 |  | 160 (4.67) | 159 (4.64) |  | -0.001 |
| Single, divorced, and windowed | 459 (13.38) | 1278 (13.29) |  | -0.003 |  | 459 (13.41) | 454 (13.26) |  | -0.004 |
| **Education level, n (%)** |  |  | **<.001** |  |  |  |  | 0.890 |  |
| Elementary school or below | 2550 (74.34) | 6412 (66.68) |  | -0.163 |  | 2544 (74.30) | 2549 (74.45) |  | 0.003 |
| Middle school or above | 880 (25.66) | 3204 (33.32) |  | 0.163 |  | 880 (25.70) | 875 (25.55) |  | -0.003 |
| **BMI, n (%)** |  |  | **<.001** |  |  |  |  | 0.611 |  |
| ＜18.5 | 271 (7.90) | 526 (5.47) |  | -0.107 |  | 265 (7.74) | 284 (8.29) |  | 0.020 |
| 18.5-23.9 | 1751 (51.05) | 4538 (47.19) |  | -0.077 |  | 1751 (51.14) | 1720 (50.23) |  | -0.018 |
| ≥24 | 1408 (41.05) | 4552 (47.34) |  | 0.126 |  | 1408 (41.12) | 1420 (41.47) |  | 0.007 |
| **Smoking, n (%)** |  |  | **<.001** |  |  |  |  | 0.658 |  |
| No | 2019 (58.86) | 5296 (55.07) |  | -0.076 |  | 2016 (58.88) | 2034 (59.40) |  | 0.011 |
| Yes | 1411 (41.14) | 4320 (44.93) |  | 0.076 |  | 1408 (41.12) | 1390 (40.60) |  | -0.011 |
| **Health status, n (%)** |  |  | **<.001** |  |  |  |  | 0.968 |  |
| Good/very good | 446 (13.00) | 2440 (25.37) |  | 0.284 |  | 446 (13.03) | 453 (13.23) |  | 0.006 |
| Fair | 1719 (50.12) | 4944 (51.41) |  | 0.026 |  | 1719 (50.20) | 1713 (50.03) |  | -0.004 |
| Poor/very poor | 1265 (36.88) | 2232 (23.21) |  | -0.324 |  | 1259 (36.77) | 1258 (36.74) |  | -0.001 |
| **Drinking, n (%)** |  |  | **<.001** |  |  |  |  | 0.753 |  |
| No | 2370 (69.10) | 6197 (64.44) |  | -0.097 |  | 2364 (69.04) | 2376 (69.39) |  | 0.008 |
| Yes | 1060 (30.90) | 3419 (35.56) |  | 0.097 |  | 1060 (30.96) | 1048 (30.61) |  | -0.008 |

Abbreviations: BMI, body mass index (calculated as weight in kilograms divided by height in meters squared); NNDSD, non-neoplastic digestive system diseases; PSM, propensity score matching; SMD, standardized mean difference. * *P*<0.05; ** *P*<0.01; ****P*<0.001.
